# The Effect of Different Tillage Methods and Nitrogen Chemical Fertilizer on Quantitative and Qualitative Characteristics of Corn

**DOI:** 10.1155/2022/7550079

**Published:** 2022-04-26

**Authors:** Reza Imani, Morteza Samdeliri, Amirabbas Mousavi Mirkalaei

**Affiliations:** Department of Agronomy, Chalous Branch, Islamic Azad University, Chalous, Iran

## Abstract

Selection of suitable tillage and application of optimal nitrogen fertilizer are essential to achieve optimal efficiency in crop management. In order to investigate the effect of tillage and nitrogen fertilizer on photosynthetic pigments and quantitative and qualitative traits of corn grain, an experiment was conducted in the research farm of Islamic Azad University, Chalous Branch, Mazandaran Province, Iran, in 2016 and 2017. Experimental factors were tillage at three levels, including no-tillage (NT), conservation tillage (MT), and conventional tillage (CT) in the main plot, and nitrogen fertilizer at four levels (no nitrogen fertilizer application and 33, 66, and 100% of the recommended amount of nitrogen fertilizer) in a subplot. The results showed that, in tillage treatments, increasing nitrogen fertilizer application increased photosynthetic pigments. Carotenoids and chlorophyll b were not significantly different in CT and MT treatments. Nitrogen and grain protein, yield components, and biological yield increased with the increasing use of recommended nitrogen fertilizer. The highest grain and biological yields in MT in the second year were observed at 11633.15 and 16644.16 kg·ha^1^, respectively. Grain and biological yield in the second year than the first year were further increased in NT and MT treatments compared to CT. Yield in MT and NT treatments improves over time. Due to the time limit in land preparation in the study area, the use of MT with 100 and 66% of the recommended nitrogen fertilizer can replace CT in the area.

## 1. Introduction

Corn is one of the most important crops globally that is of great importance in human nutrition as well as animal feed [1, 2]. According to the FAO, 202 million hectares of the world's land in 2020 is devoted to corn cultivation, of which more than 1,162 million tons was produced in 2020. Also in Iran, the area under corn cultivation is 205 thousand hectares, and its production is 1.4 million tons [3].

Despite the large corn production level, the average yield of corn has not yet reached its genetic potential. In addition to innovations in breeding technology, some cropping methods such as crop rotation, tillage, and fertilization also need to be improved [4, 5]. Tillage methods are used as basic and important operations to achieve higher crop yields [6, 7]. Therefore, tillage is one of the essential operations for successful corn production. Tillage systems can significantly affect the yield and nutritional quality of maize by affecting temperature, humidity, aeration, and access to food [5]. However, frequent use of machinery and tillage operations at certain depths is one of the main reasons for soil compaction. Different tillage operations affect soil compaction because the production of crops NT for many years affects access to food (due to the formation of a hard layer in the substrate) [8, 9]. In this case, a gradual increase in soil density reduces nitrogen uptake and ultimately reduces corn grain quality [9]. Soil density has been reported to reduce the uptake of nitrogen (11–15%), phosphorus (11–15%), and potassium (5–10%) in wheat [10].

Reducing soil nitrogen uptake by plants has a great impact on crop growth and yield because nitrogen is one of the most important nutrients for plants, especially corn, which, if not consumed in sufficient quantities, will limit the growth of plant growth [9]. Due to various climatic and soil factors such as nutrient status, soil properties, and the reaction of cultivated nutrients, maize responds positively to nitrogen application and increases dry matter production [9, 11, 12]. Nitrogen consumption improves the yield components of maize [13] as it has been reported that nitrogen application leads to increased biomass production (22%) and grain yield (24%) [14]. On the other hand, it has been reported that, with higher consumption of nitrogen in the soil, crop production is negatively affected [15]. Also, higher amounts of nitrogen can cause environmental problems, such as nitrate leaching, excessive nutrients in any water body, and greenhouse gas emissions [16]. Therefore, it is important to study the use of nitrogen fertilizer to grow and reduce environmental hazards in the region.

Tillage and nitrogen fertilizer are significantly correlated [17]. Several researchers studied the response of crop yield to nitrogen fertilizer under different tillage methods [18–20]. The results of a five-year study of the effect of nitrogen application and tillage showed that corn grain yield in CT (moldboard plow) is higher than treatment without tillage. The researchers also reported that, in both tillage systems, increasing nitrogen fertilizer application had a significant effect on maize dry matter yield [17]. The effect of tillage on corn yield is very variable. As reported in some studies, no difference in corn yield was observed between MT and CT [21, 22]. In some studies, a decrease in maize yield in MT has been reported compared to CT [23–25]. However, there are many reports of positive effects and improvement of soil properties and yield in MT on maize [26, 27]. Studies by Kihara and Bationo [28] reported that the NT system yield was more than CT, which is achieved over several seasons. In the method NT compared to other methods, less residue is mixed with the soil, and therefore residue decomposition and nitrogen release are slow. Hence, in order to achieve equal yield with the CT method, it is necessary to increase the amount of nitrogen fertilizer in the first years of the NT method [29]. Consequently, proper use of nitrogen is essential for crop yield growth and decomposition of crop residues in tillage methods.

Research on growth and yield in relation to the tillage system under different levels of nitrogen fertilizer for the current area has not been conducted. Also, for crop management, it is necessary to choose the most suitable tillage method and the amount of nitrogen fertilizer due to time limit. Therefore, the purpose of this experiment was to investigate different tillage systems and nitrogen fertilizer levels on quantitative and qualitative traits of maize.

## 2. Materials and Methods

### 2.1. Study Site and Soil Physicochemical Analyses

This experiment was carried out during two cropping years of 2016 and 2017 in the Islamic Azad University research farm, Chalus Branch. The experiment site is located at latitude 40° 58ˊ north, longitude 53° 69ˊ east, and altitude 3 m above sea level. Based on soil test from depth of 0 to 30 cm, soil texture (sandy-clay-loam), electrical conductivity (1.34 dS m1), pH (7.1), organic carbon (0.9%), nitrogen (0.08%), phosphorus (11 ppm), and potassium (314 ppm) were determined.

### 2.2. Experimental Design

The experiment was performed as a split-plot in a randomized complete block design with three replications. Experimental factors include tillage at three levels (no-tillage (NT), conservation tillage (MT), and conventional tillage (CT)) in the main plot and sources of nitrogen fertilizer at four levels, including nonapplication of zero, 33, 66, and 100% of the recommended amount of nitrogen fertilizer based on soil test in the subplot. Each plot consisted of six planting rows, the distance between planting rows was 75 cm, and the plant spacing on the row was 20 cm. The distance between the main plots was three meters and between each replication was 4 m. The seeds were planted in early May according to the regional weather conditions. Minimum and maximum temperature, relative humidity, and precipitation of the experimental site are presented in Table 1.

### 2.3. Cultivation Practices

CT was done with moldboard plow (with a depth of 20–25 cm) + disc (2 times with a depth of 15–10 cm) + leveler + drill planter, MT used a combination cultivator tillage machines + drill planter, and NT treatment did not use machinery, only sowing the seeds with drill planter. The application rate of 100% nitrogen fertilizer (source of urea fertilizer) was equivalent to 300 kg ha^−1^. Also, 66 and 33% of nitrogen fertilizer application was equivalent to 198 and 99 kg ha^−1^, respectively. Based on the water requirement of corn in the climatic conditions of the region and the soil conditions of the field, the irrigation schedule of the area was adjusted based on meeting the needs of the plant and preventing the occurrence of moisture stress.

In order to achieve proper density, the plant was thinned in one stage after complete establishment in the four-leaf stage. Nitrogen fertilizer from urea fertilizer source was applied to the ground in three stages (planting, stemming, and flowering) and phosphorus fertilizer from triple superphosphate source before planting. According to the soil test results, there was no need for potash fertilizer.

### 2.4. Measurements

Harvest was done in late August. To determine the yield and yield components, by removing the side rows and 50 cm from the beginning and end of each plot as a margin effect, 1.05 m^2^ of each plot's middle part was chosen and transferred to the laboratory. Then, 1000-grain weight, number of grains per row, number of rows per ear, biological yield, and grain yield were measured. The content of grain nitrogen was measured by titration after distillation using an automatic device (Tecator Kjeltec Auto 10 analyzer) [30]. Measurement of chlorophyll a and b in young leaves of each treatment was performed by the method of Arnon [31]. Also, the method of [32] was used to measure carotenoids.

### 2.5. Statistical Analysis

Analysis of data variance was done with SAS v.3 software, and Duncan's multiple range tests were used at a probability level of 5% to compare the mean of the desired traits. In this experiment, the effect of the year was considered random.

## 3. Results

### 3.1. Photosynthetic Pigments

#### 3.1.1. Chlorophyll a

The results of the variance analysis of the effect of tillage and nitrogen fertilizer on photosynthetic pigments are shown in Table 2. The results showed that the effect of nitrogen fertilizer and the interaction of tillage × nitrogen and year × tillage on chlorophyll a were significant (Table 2). The highest chlorophyll a in CT and MT treatments in the second year was 1.19 and 1.18 mg·g^−1^ FW, respectively. The lowest amount was observed in NT treatment in the first year, with 0.75 mg·g^−1^ FW. Chlorophyll a increased in all three tillage treatments in the second year compared to the first year, but chlorophyll a in the second year than the first year had a further increase in NT (31.82%) treatments compared to CT (10.17%) and MT (8.40%) (Table 3). 

**Figure 1:**
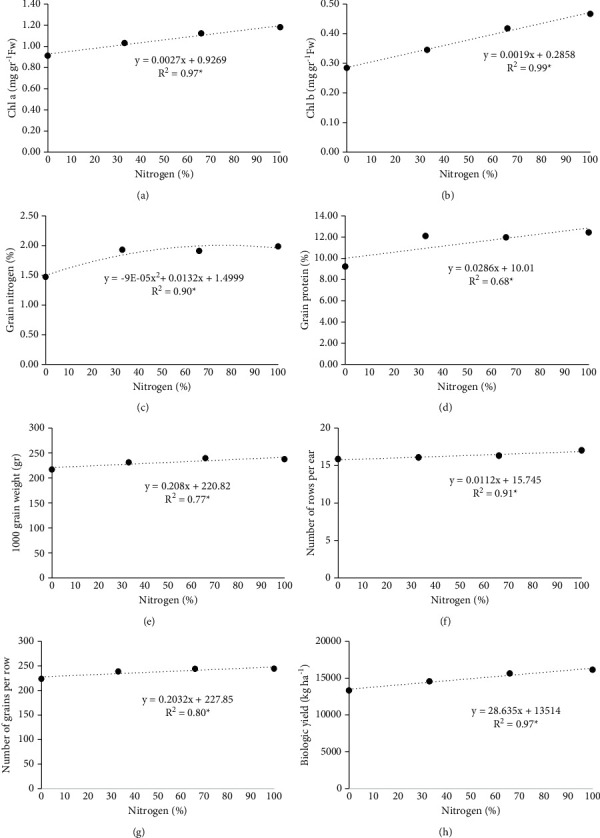
The trend of changes in nitrogen fertilizer levels on chlorophyll a (a), chlorophyll b (b), grain nitrogen (c), grain protein (d), 1000-grain weight (e), number of rows per ear (f), number of grains per row (g), and biological yield (h).

**Figure 2:**
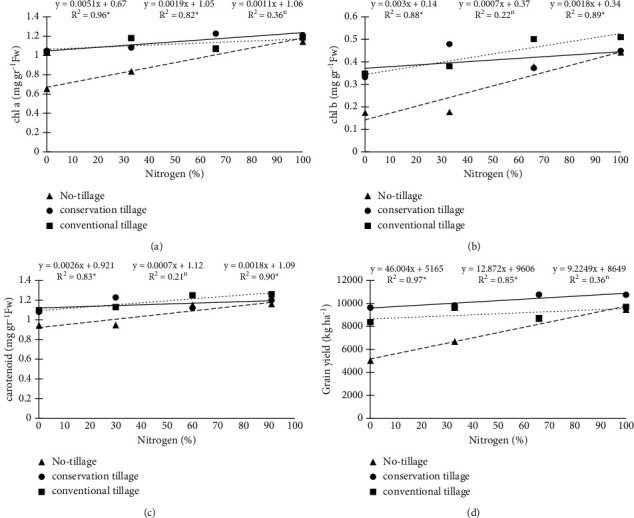
The trend of nitrogen fertilizer changes on chlorophyll a (a), chlorophyll b (b), carotenoids (c), and grain yield (d) of corn under tillage treatments.

The chlorophyll a change trends under the influence of nitrogen fertilizer are shown in Figure 1(a). The results showed that, with increasing nitrogen fertilizer, chlorophyll a increased so that, with increasing one unit of nitrogen fertilizer, chlorophyll a increased by 0.0027 mg·g^−1^ FW. The highest chlorophyll a was obtained in the treatment of 100% nitrogen fertilizer at the rate of 1.18 mg·g^1^ FW (Figure 1(a)). Also, the trend of chlorophyll a change in tillage treatments showed that chlorophyll a had a significant increase with increasing nitrogen fertilizer in MT and NT treatments and showed a nonsignificant increasing trend in CT. In MT and NT, a unit increase of nitrogen fertilizer increased 0.0019 and 0.0051 mg g^1^ FW of chlorophyll a, respectively. Due to the slope of the line, the rate of increase of chlorophyll a in treatments NT and then MT was more than CT. The highest chlorophyll a value was obtained in MT and 66% nitrogen fertilizer (Figure 2(a)).

#### 3.1.2. Chlorophyll b

**Figure 3:**
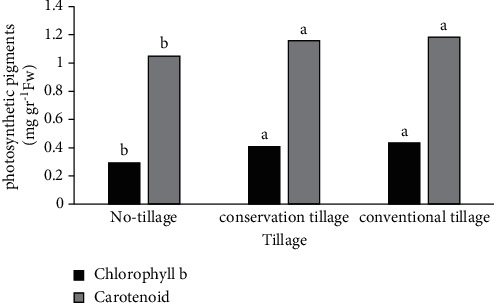
The effect of tillage methods on chlorophyll b and carotenoids.

According to the variance analysis results, tillage and nitrogen fertilizer and their interaction on chlorophyll b had a significant effect (Table 2). Chlorophyll b was affected by nitrogen fertilizer, and with increasing nitrogen fertilizer, chlorophyll b increased. Also, by raising one nitrogen fertilizer unit, chlorophyll b increased by 0.042 mg·g^−1^ FW. The highest chlorophyll b value was obtained in the treatment of 100% nitrogen fertilizer at the rate of 0.47 mg·g^−1^ FW (Figure 1(b)). The trend of chlorophyll b changes under the influence of nitrogen fertilizer in tillage treatments is shown in Figure 2(b). Chlorophyll b increased significantly with increasing nitrogen fertilizer in CT and NT treatments and showed a nonsignificant increasing trend in MT. In CT and NT, one unit increase of nitrogen fertilizer increased 0.0018 and 0.003 mg·g^1^ FW of chlorophyll b, respectively. The highest chlorophyll b was obtained in 100% nitrogen fertilizer treatment and CT (0.51 mg·g^−1^ FW) (Figure 2(b)). The results showed that the highest chlorophyll b was obtained in CT and MT treatment at the rate of 0.43 and 0.41 mg·g^−1^ FW, and the lowest value was observed in the treatment NT (Figure 3).

### 3.2. Carotenoids

The results of the analysis of variance showed that the main effect of tillage and also the interaction of nitrogen × tillage on carotenoids were significant (Table 2). Carotenoids increased significantly with increasing nitrogen fertilizer in CT and NT treatments and showed a nonsignificant increase in MT. In CT and NT, one unit increase of nitrogen fertilizer increased 0.0018 and 0.0026 mg g^1^ FW of carotenoids, respectively (Figure 2(c)). It seems that the use of nitrogen has increased the root and leaf growth of the plant, which has led to an increase in carotenoids in the plant. CT and MT treatments had the highest amount of carotenoids at 1.18 and 1.16 mg·g^−1^ FW, respectively. The lowest amount was observed in NT treatment (Figure 3). It can be inferred that the existence of suitable substrate conditions in CT and rapid establishment of the plant to exploit the growing season leads to enhanced growth and increases plant uptake. These factors can eventually increase the density of pigments (including carotenoids) per unit leaf area.

### 3.3. Qualitative Traits

#### 3.3.1. Grain Nitrogen

The results of an analysis of variance of tillage and nitrogen fertilizer effects on grain nitrogen are shown in Table 2. The results showed that the main effect of nitrogen fertilizer on grain nitrogen was significant (Table 2). The results of the nitrogen fertilizer effect showed that, with increasing nitrogen fertilizer, grain nitrogen almost increased. Growing nitrogen fertilizer from 0 to 66%, the slope of grain nitrogen increase was higher, and with increasing nitrogen fertilizer to 100%, the slope of grain nitrogen increase was lower (Figure 1(c)).

#### 3.3.2. Grain Protein

The results showed that the effect of nitrogen fertilizer on grain protein was significant (Table 2). The trend of grain protein changes under the influence of nitrogen fertilizer is shown in Figure 1(d). The results showed that, with increasing nitrogen fertilizer, grain protein increased so that, with increasing one unit of nitrogen fertilizer, grain protein increased by 0.028%. The highest grain protein is obtained from the treatment of %100 nitrogen fertilizers (Figure 1(d)).

### 3.4. Yield and Yield Components

#### 3.4.1. Number of Rows per Ear

The results of the analysis of variance showed that the nitrogen fertilizer and the interaction of year × tillage had a significant effect on the number of rows per ear (Table 4). The highest number of rows per ear in MT treatment in the second year was 17 rows, and the lowest was observed in the treatment NT in the first year of 14 rows. The number of rows per ear in NT and MT treatments increased by 11.53% and 3.57% in the second year compared to the first year (Table 3).

The results show that, with increasing nitrogen fertilizer, the number of rows per ear has increased. Increasing one unit of nitrogen fertilizer, the number of rows per ear increases by 0.011 rows. The highest number of rows per ear was obtained in the treatment of 100% nitrogen fertilizer with 17 rows, and the lowest was observed in the treatment without the use of nitrogen fertilizer (Figure 1(f)).

#### 3.4.2. Number of Grains per Ear

According to the variance analysis, the nitrogen fertilizer and the interaction of the year × tillage had a significant effect on the number of grains per ear (Table 4). The highest number of grains per ear was obtained in MT treatment in the first and second years and CT in the first year (Table 3).

The trend of changes in the number of grains per ear under the influence of nitrogen fertilizer is shown in Figure 1(g). The results showed that, with increasing nitrogen fertilizer, the number of grains per ear increased. With increasing one unit of nitrogen fertilizer, the number of seeds per ear increased by 0.203 grains. The highest number of grains per ear was obtained in the treatment of 100 and 66% of nitrogen fertilizer with 245 and 244 grains (Figure 1(g)).

#### 3.4.3. 1000-Grain Weight

The results showed that the nitrogen fertilizer and the interaction of the year × tillage had a significant effect on 1000-grain weight at the level of 1% probability (Table 4). The highest 1000-grain weight in CT treatment in the first year was 253.15 g, and the lowest in NT treatment in the first year was 212.60 g (Table 3). In CT and MT, increasing the level of plant uptake, which includes the uptake of nutrients and ions required, conserving photosynthetic sources during the growing season, receiving radiant energy, and transferring photosynthetic material to the grain, increases the total weight of 1000 grains. 1000-grain weight in MT and NT treatments increased in the second year compared to the first year (Table 3).

The trend of 1000-grain weight changes under the influence of nitrogen fertilizer is shown in Figure 1(e). The results showed that, with increasing nitrogen fertilizer, the weight of 1000 grains increased so that, with increasing one unit of nitrogen fertilizer, the weight of 1000 grains increased by 0.208 g. The weight of 1000 grains increases significantly with the application of fertilizer compared to its nonapplication.

### 3.5. Grain Yield

The results of the analysis of variance of tillage and nitrogen fertilizer on yield traits are shown in Table 4. The results showed that the interaction effect of tillage × nitrogen and year × tillage on grain yield was significant (Table 4). The highest grain yield was observed in MT treatment in the second year at the rate of 11633.15 kg ha^1^, and the lowest was in the NT treatment in the first year. Grain yield in all three tillage treatments increased in the second year compared to the first year, but grain yield in the second year than the first year was further increased in NT (34.75%) and MT (23.84%) treatments compared to CT (11.10%) (Table 3).

**Figure 4:**
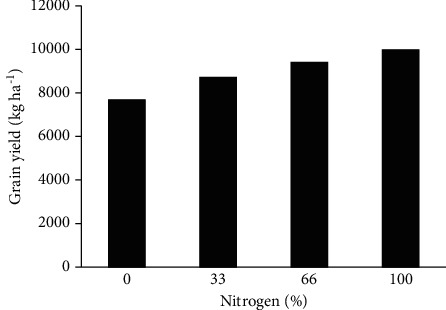
Effect of nitrogen fertilizer on grain yield of corn.

The trend of grain yield changes in tillage treatments showed that grain yield increased significantly with increasing nitrogen fertilizer in MT and NT treatments and showed a nonsignificant increase in CT (Figure 2(d)). In MT and NT, one unit of nitrogen fertilizer increased and grain yield increased by 12.87 and 46.00 kg·ha^1^, respectively. Due to the line's slope, the increase rate in grain yield in treatments NT and then MT was higher than CT. The highest grain yield was obtained in MT and 66 and 100% nitrogen fertilizer (Figure 2(d)). The results showed that grain yield increased nonsignificantly with increasing the use of nitrogen fertilizer. The use of 100% recommended nitrogen fertilizers was 2.3 t·ha^−1^ compared to nonuse (Figure 4).

### 3.6. Biological Yield

This study showed that the nitrogen fertilizer and the interaction of the year × tillage had a significant effect on biological yield (Table 4). The highest biological yield in MT treatment in the second year was 16644.16 kg·ha^1^. Biological yield in MT and NT treatments increased in the second year compared to the first year (Table 3).

The results of the trend of biological yield changes under the influence of nitrogen fertilizer showed that, with the increase of nitrogen fertilizer, biological yield increased. With the increase of one unit of nitrogen fertilizer, biological yield increases to 28.63 kg·ha^−1^. The highest biological yield was obtained in 100% nitrogen fertilizer treatment, by 16170.80 kg·ha^−1^ (Figure 1(h)).

## 4. Discussion

Photosynthetic pigments were higher in CT treatment than in other treatments. It can be inferred that the existence of suitable substrate conditions in CT and rapid establishment of the plant to exploit the growing season leads to enhanced growth and increases plant uptake. These factors can eventually increase the density of pigments per unit leaf area. Photosynthetic pigments in MT than in CT treatment were slightly different. Photosynthetic pigments have increased in MT tillage treatment due to plowing and plant growth conditions, but in NT treatment due to soil compaction and lack of plant root development, growth conditions were not provided, so photosynthetic pigments were low in the first year. The further increase of photosynthetic pigments in the second year compared to the first year in the NT treatment was due to the fact that, in the second year, the improvement of environmental conditions, including decreasing soil density and increasing soil organic matter in NT treatment, led to improved growth and chlorophyll content.

The amount of photosynthetic pigments decreased with decreasing nitrogen fertilizer application. The chlorophyll in chloroplasts cannot synthesize without the presence or lack of nitrogen, and the photosynthetic and chlorophyll activities are reduced or stopped. Nitrogen deficiency, due to reduced size and durability of leaf area, reduces the amount of radiation received and radiation use efficiency [33]; as a result, the photosynthesis of the crop is reduced. It seems that the use of nitrogen has increased the root and leaf growth of the plant, which has led to an increase in photosynthetic pigments in the plant. Ciompi and Gentili [33] reported that reducing nitrogen in the plant decreases the chlorophyll a content.

Nitrogen fertilizer caused an increase in nitrogen and protein content of grain. Increasing nitrogen fertilizer to meet the corn plant's nutritional needs increases the photosynthetic capacity of the corn plant. On the other hand, corn has strong roots and can also absorb nutrients from the soil [34]. It seems that, by adding chemical fertilizer to the soil, the amount of soil nitrogen increased, consequently, the amount of uptake of this element by the plant increased, and by transferring it to the grain, the content of grain nitrogen increased. Also, nitrogen is the main constituent of protein structure, which probably increases the grain nitrogen storage using a nitrogen fertilizer. With increasing the amount of this element, the content of grain protein increased. The positive role of nitrogen in increasing grain protein content has been reported by researchers [35].

The number of rows per ear in NT and MT treatments increased in the second year compared to the first year. Increasing the number of rows per ear in MT and NT treatments in the second year of the experiment can be due to improved aggregate stability, increased soil organic matter, higher soil water permeability, and overall better environmental growth conditions [36,37]. These factors have increased the number of rows in the second year of the experiment. Reinbott and Conley [38] also stated about corn and grain sorghum that the highest number of seeds and rows was observed in MT treatment and the lowest was in NT treatment.

The highest number of grains per row was MT and CT. According to the results, it can be said that the amount of photosynthetic pigments in MT and CT is higher than in NT, which has led to improved growth and an increased number of grains per plant. In general, tillage affecting soil mechanical strength, soil aeration, cohesion and stability, pore size, and the amount of soil pores, soil temperature, soil water content, soil nutrients, and their interaction can affect root growth amount. As a result, it affects the growth of the Shoot of the plant. Reinbott and Conley [38] also stated about corn and grain sorghum that the highest number of seeds was observed in MT treatment. The lowest was in NT treatment, which is consistent with the results of this study. The study of tillage systems on spring barley has shown that the reduction of tillage levels leads to a decrease in the number of grains per spike, and the highest number of grains per spike was obtained in the CT system (plow + disc) [39].

In CT and MT, increasing the level of plant uptake, which includes the uptake of nutrients and ions required, conserving photosynthetic sources during the growing season, receiving radiant energy, and transferring photosynthetic material to the grain, increases the total weight of 1000 grains. Improved growth conditions due to reduced soil density in the second year are one reason that the weight of 1000 grains improved in the second year. It seems that one of the reasons for the reduction of 1000-grain weight in NT treatment was the decrease in biological yield and consequently the low photosynthetic levels at the time of grain filling. In separate experiments, it was observed that the weight of 1000 grains of sunflower and corn in the NT system was reduced compared to the CT system, while there was no statistically significant difference between the MT and CT system [40].

By increasing the use of nitrogen fertilizer from zero to 100% of the recommended amount, corn yield components increased. It seems that increasing the use of nitrogen fertilizer in addition to removing nitrogen restrictions for corn increases photosynthetic and plant production efficiency and ultimately leads to an increase in the yield components. It can be seen that these results were consistent with the findings of Mandal and Das [41]. Similar results have been reported by Reed and Singletary [42] and Prasad and Singh [43] to increase the number of grains per ear in proportion to the increase in chemical fertilizer levels. Hamidi and Dabbagh Mohammadi Nasab [44] reported that the availability of nutrients, especially nitrogen, in the critical period of seed formation by increasing plant growth affects the number of grains. Treatment without nitrogen fertilizer reduced the number of grains per ear. The cause of grain loss in nitrogen deficiency conditions may be infertility, increased abortion, or underdevelopment. Moser and Feil [45] stated that corn without nitrogen fertilizer produces fewer rows of grain per ear, which is consistent with this study's results.

The high efficiency of corn is of particular importance in applying nitrogen, which is due to the creation of an efficient photosynthetic system of corn. Therefore, the increase in 1000-grain weight is due to the increase in photosynthesis intensity and the transfer of nutrients to the seeds. Increasing the use of chemical fertilizers removes food restrictions for corn, increases the plant's photosynthetic and production efficiency, and ultimately increases the weight of 1000 grains. Turk and Tawaha [46] also showed that beans respond well to different levels of fertilizer. The weight of 1000 grains increases significantly with the application of fertilizer compared to its nonapplication.

Grain yield increased in the second year compared to the first year in all treatments. According to meteorological data (Table 1), the average minimum and maximum temperatures in the first year of the experiment were 19.55 and 27.42°C, respectively, and in the second year were 22.94 and 28.69°C, respectively. The total precipitation in the first and second years was 37.76 and 41.1 mm, respectively. Therefore, the optimal growth temperature of corn, especially in the early stages of growth, can be considered the reason for the higher grain yield in the second year. Also, high rainfall and its proper distribution during the plant growth period in the second year is one of the reasons for increasing yield.

The high number of rows per ear and the number of grains per row in the MT treatment have led to increased grain yield. Increased grain yield under the influence of MT has also been reported by Zhang and Cao [47]. Singer and Kohler [22] reported that selecting the appropriate tillage method and preparing the substrate ultimately affects crop yield. On the other hand, Schillinger and Young [48] found that MT can be equal to or even better in yield than CT. In another study, it was stated that crop yield in MT improved because MT increased the organic matter in the soil surface [49].

The lowest grain and biologic yield were observed in the treatment without tillage in the first year. Increasing soil compaction and lack of suitable conditions for root growth are the reasons for the reduced production in the system without tillage. This compaction can reduce root length and ultimately reduce the uptake of water and nutrients by the plant, resulting in a uniform growth in the NT method in the field, leading to a decrease in yield. Similar results have been reported in other studies [50–52]. Studies by Botta and Tolón-Becerra [53] and Kwaw‐Mensah and Al‐Kaisi [54] showed that increasing soil compaction is a barrier to plant growth and thus affects yield.

The highest grain yield was observed in conservation tillage in the maximum amount of recommended nitrogen fertilizer. Alvarez and Steinbach [55] and Halvorson and Mosier [17] stated that corn yield increased with nitrogen fertilizer application in MT in a long-term study of tillage systems. It has been well shown that grain yield and biological yield of crops respond positively to nitrogen fertilizer. Due to their essential role in increasing plant growth, high consumption elements, especially nitrogen, increase plant yield. On the other hand, the increased power of corn in absorbing these elements is of particular importance, which is due to the efficient photosynthetic system of corn. Therefore, increasing the amount of fertilizer by increasing the number of seeds and the weight of 1000 grains indirectly increases the yield in MT treatment. Also, in this study, by consuming higher nitrogen fertilizers, the uptake and transfer of this element to different parts of the plant increased. Scharf and Wiebold [56] reported that nitrogen fertilizer consumption increased the biological yield of maize, which is consistent with the results of this study. Studies show that the biological yield of crops responds positively to nitrogen fertilizer [17]. On the other hand, soil nitrogen availability during the seedling stages of maize has a decisive role in the growth and yield of maize. As nitrogen plays an essential role in increasing the plant's vegetative growth, it ultimately increases plant yield. Increasing the greenery growth provides more leaf area for the plant, which can produce more dry matter [57].

## 5. Conclusion

The results of this study showed that yield and grain yield components in tillage treatments increased in the second year compared to the first year, which was a further increase in NT and MT treatments. One of the reasons for this increase in these two treatments is the improvement of environmental conditions and reduction of soil density by increasing organic matter in the second year, which has led to improved root and plant growth and development. In general, the use of MT is appropriate because of its advantages over CT in the study area.

The trend of changes in nitrogen fertilizer application showed that, with increasing nitrogen fertilizer, the measured traits increased. Also, the results of the interaction of tillage and nitrogen showed that grain yield increased significantly with increasing nitrogen fertilizer in MT and NT treatments. Due to the slope of the regression line, the rate of increase in chlorophyll a and grain yield in treatments without tillage and then MT was higher than CT. Therefore, according to grain yield, the most appropriate treatment for the study area is the use of MT in the conditions of using 100 and 66% nitrogen fertilizer.

## Figures and Tables

**Table 1:** Meteorological parameters for the field site during the experiment (Mazandaran Province Meteorological Office).

Months	Minimum temperature (°C)		Maximum temperature (°C)		Relative humidity (%)		Precipitation (mm)	
	2016	2017	2016	2017	2016	2017	2016	2017
May	13.3	16.7	22.6	23.4	76	78	26.3	23.9
June	20.3	23.56	26.4	28.28	82	83	43.25	46.5
July	24.5	27.6	28.6	29.7	79	78	35.3	38.4
August	20.1	23.9	32.1	33.4	85	88	46.2	55.6

**Table 2:** Variance analysis of photosynthetic pigments of nitrogen and protein grain of corn in different tillage and nitrogen levels for two years.

SOV	df	Chl a	Chl b	Carotenoid	Grain nitrogen	Grain protein
Year (Y)	1	0.672^*∗∗*^	0.192^*∗∗*^	0.163^*∗∗*^	0.002 ^ns^	0.10 ^ns^
Block (year)	4	0.011	0.001	0.002	0.095	3.72
Tillage (T)	2	0.341 ^ns^	0.137^*∗*^	0.122^*∗*^	0.314 ^ns^	12.36 ^ns^
Y x T	2	0.112^*∗*^	0.001 ^ns^	0.001 ^ns^	0.043 ^ns^	1.69 ^ns^
Error tillage	8	0.019	0.005	0.006	0.057	2.22
Nitrogen (N)	3	0.447 ^*∗*^	0.116^*∗*^	0.099 ^ns^	1.013^*∗∗*^	39.83^*∗∗*^
Y x N	3	0.043 ^ns^	0.008 ^ns^	0.014 ^ns^	0.003 ^ns^	0.11 ^ns^
T x N	6	0.068^*∗*^	0.034^*∗∗*^	0.029^*∗∗*^	0.063 ^ns^	2.47 ^ns^
Y x T x N	6	0.021 ^ns^	0.008 ^ns^	0.009 ^ns^	0.028 ^ns^	1.09 ^ns^
Error total	36	0.021	0.007	0.006	0.077	3.04

^ns^, ^*∗*^, and ^*∗∗*^: not significant and significant at the 5% and 1% probability levels, respectively.

**Table 3:** Comparison mean the effect of different tillage on quantitative and qualitative traits of maize in two years.

Tillage	Year	Chl a (mg gr^1^ Fw)	Number of grains per ear	Number of rows per ear	1000-grain weight (gr)	Grain yield (kg·ha^1^)	Biologic yield (kg·ha^1^)
NT	2016	0.75 d	225.10 c	14.65 c	212.60 d	5886.18 d	12548.82 c
	2017	1.10 b	224.67 c	16.56 b	238.15 b	9021.31 bc	14665.76 b
MT	2016	1.09 b	250.32 a	16.46 b	227.58 c	8860.25 c	14864.73 b
	2017	1.19 a	247.16 a	17.07 a	237.32 b	11633.15 a	16644.17 a
CT	2016	1.06 c	230.27 b	16.65 b	253.15 a	8573.12 c	15874.54 ab
	2017	1.18 a	250.21 a	16.44 b	218.21 d	9643.26 b	15034.54 b

According to Duncan's multiple range test, means with the same letters in each column are not significantly different.

**Table 4:** Analysis of variance of yield and yield components of maize in different levels of tillage and nitrogen in two years.

SOV	df	Number of grains per ear	Number of rows per ear	1000-grain weight	Grain yield	Biologic yield
Year (Y)	1	534.85 ^ns^	10.64^*∗∗*^	0.23 ^ns^	97389356.12^*∗∗*^	18682845.09^*∗*^
Block (year)	4	149.34	0.46	66.48	803603.3678	1719833.91
Tillage (T)	2	3508.04 ^ns^	9.11 ^ns^	667.07 ^ns^	47336028 ^ns^	32450269.29 ^ns^
Y × T	2	956.37^*∗∗*^	6.83^*∗∗*^	5904.49^*∗∗*^	7294767.238^*∗*^	15718803.16^*∗∗*^
Error tillage	8	46.73	0.43	34.85	1434117.537	717108.26
Nitrogen (N)	3	1708.00^*∗*^	39.64^*∗*^	1861.21^*∗*^	17508121.25 ^ns^	28173846.67^*∗*^
Y × N	3	96.75 ^ns^	0.81 ^ns^	118.01 ^ns^	3866566.103 ^ns^	1599220.12 ^ns^
T × N	6	333.63 ^ns^	2.83 ^ns^	155.93 ^ns^	5723898.047^*∗*^	2520070.68 ^ns^
Y × T × N	6	159.98 ^ns^	1.85 ^ns^	54.45 ^ns^	1243062.003 ^ns^	1082281.22 ^ns^
Error total	36	81.43	0.92	73.75	2271529.509	971234.78

^ns^, ^*∗*^, and ^*∗∗*^: not significant and significant at the 5% and 1% probability levels, respectively.

## Data Availability

The data used to support the findings of this study are available from the corresponding author upon request.
